# Transcriptional and DNA Methylation Signatures of Subcutaneous Adipose Tissue and Adipose-Derived Stem Cells in PCOS Women

**DOI:** 10.3390/cells11050848

**Published:** 2022-03-01

**Authors:** Adeline Divoux, Edina Erdos, Katie Whytock, Timothy F. Osborne, Steven R. Smith

**Affiliations:** 1 Translational Research Institute, AdventHealth, Orlando, FL 32804, USA; katie.whytock@adventhealth.com (K.W.); steven.r.smith@adventhealth.com (S.R.S.); 2 Departments of Medicine and Biological Chemistry, Division of Diabetes Endocrinology and Metabolism, Institute for Fundamental Biomedical Research, Pediatrics Johns Hopkins University School of Medicine, Johns Hopkins All Children’s Hospital, St. Petersburg, FL 33701, USA; eerdos@jhmi.edu (E.E.); tosborn9@jh.edu (T.F.O.)

**Keywords:** PCOS, adipose tissue, gluteofemoral, adipose-derived stem cell, transcriptome, DNA methylation

## Abstract

Polycystic ovary syndrome (PCOS) is often associated with metabolic syndrome features, including central obesity, suggesting that adipose tissue (AT) is a key organ in PCOS pathobiology. In this study, we compared both abdominal (ABD) and gluteofemoral (GF) subcutaneous AT in women with and without PCOS. ABD and GF subcutaneous ATs from PCOS and BMI/WHR-matched control women were analyzed by RT-qPCR, FACS and histology. ABD and GF adipose-derived stem cell (ASC) transcriptome and methylome were analyzed by RNA-seq and DNA methylation array. Similar to the control group with abdominal obesity, the GF AT of PCOS women showed lower expression of genes involved in lipid accumulation and angiogenesis compared to ABD depot. FACS analysis revealed an increase in preadipocytes number in both AT depots from PCOS. Further pathway analysis of RNA-seq comparisons demonstrated that the ASCs derived from PCOS are pro-inflammatory and exhibit a hypoxic signature in the ABD depot and have lower expression of adipogenic genes in GF depot. We also found a higher CpG methylation level in PCOS compared to control exclusively in GF-ASCs. Our data suggest that ASCs play an important role in the etiology of PCOS, potentially by limiting expansion of the healthy lower-body AT.

## 1. Introduction

Polycystic ovary syndrome (PCOS) is a complex condition characterized by ovulatory dysfunction and androgen excess that affects 12–18% of reproductive-age women, depending on the diagnostic criteria used [1]. Women with PCOS have an increased risk for dyslipidemia, an important risk factor for type 2 diabetes mellitus (T2D) and an overall increased prevalence of metabolic syndrome [2,3]. Moreover, insulin resistance (IR) prevalence rates are between 44 and 85% in this specific population [4], the variability observed being in part due to differences in PCOS phenotype and ethnicity [5]. In addition, PCOS women are more likely to be overweight and have central obesity compared to the general population [6], suggesting that adipose tissue (AT) distribution is part of the syndrome. Several studies confirmed the association with central obesity by emphasizing AT alterations in PCOS women such as hypertrophic adipocytes [7], impairments in lipolysis and insulin action [8] and dysregulation of adipokine expression and secretion implicated in IR [9]. Women with PCOS were found to also have multiple transcriptional and DNA methylation modifications in their abdominal (ABD) AT [10]. However, in the majority of the studies, PCOS women were confounded by a higher BMI, waist to hip ratio (WHR) or HOMA-IR compared to the control group. Nevertheless, researchers have shown that obesity and PCOS have both a separate and synergistic relationship with IR and other determinants of metabolic syndrome [11].

The concept that obesity-related comorbidities are, in large part, originated in the AT itself is now well established [12,13,14]. During development of obesity, AT acquires a chronic inflammatory state associated with release of cytokines and chemokines [15] along with a potential accumulation of fibrosis [16,17,18]. Another common hypothesis states that once the AT reaches its limited capacity for expansion, lipids begin to accumulate in other tissues [19,20,21]. An extension of this theory is that the lower-body or gluteofemoral (GF) fat is the optimal depot to store lipids and any factor limiting its expansion leads to sequential accumulation of energy in the upper-body AT depots (ABD and visceral) and eventually spills over into other organs, such as liver [22]. This could explain why a predominantly upper-body fat distribution, commonly associated with higher visceral fat, is associated with increased risk factors for CVD, T2D and PCOS [23], whereas favorable lower-body fat distribution seems protective against these risks [24]. Prior work in our lab supported this hypothesis by showing that GF adipocytes from pear-shaped women (characterized by low WHR) expressed more genes involved in lipid and glucose metabolism compared to adipocytes isolated from the same fat depot but from apple-shaped women (characterized by high WHR) [25]. Interestingly, there are no reported studies exploring the lower-body adipose depot in PCOS.

Here, we report a study of ABD and GF AT depots from PCOS women and compare their characteristics to the same fat depots isolated from non-PCOS women. Importantly, both groups of women had similar BMI and WHR, all women being obese, and accumulated fat preferentially in the abdominal depot (apple-shaped). We tested the hypothesis that PCOS women have a limited capacity to store in their GF AT depot, leading to fat accumulation in their ABD depots (subcutaneous and visceral). We investigated gene expression and secretome profiles of the ABD and GF subcutaneous fat depots, along with their cellular content using cytometric analysis. We also isolated, cultured and studied adipose-derived stem cells (ASCs) from both subcutaneous AT depots using RNA-seq and DNA methylation arrays. Our results support our a priori hypothesis that, compared to matched upper-body obese premenopausal control women, the GF subcutaneous ATs of PCOS women have limited expansion capacity characterized by abnormal ASC transcriptome and methylome. These findings reveal a fundamental role for the metabolic features of AT in the phenotypic complications of PCOS.

## 2. Materials and Methods

### 2.1. Participants

Nineteen healthy premenopausal, weight-stable females aged 20–40 years with a body mass index (BMI) between 31 and 40 kg/m^2^ were recruited in Orlando using advertisements approved by an institutional review board. Subjects were excluded if they reported a history of chronic disease (diabetes, heart or liver disease, high blood pressure, and gastrointestinal disorder), recent weight loss or gain (>3 kg over the past 8 weeks), had abnormal blood or urine and clinical lab values, or used oral contraceptives or hormone replacement therapy. PCOS was diagnosed based on NIH criteria [26]. Abdominal (ABD) AT biopsies were collected with a three-hole 2.5 mm liposuction cannula from the mid-abdomen approximately 5–8 cm lateral to the umbilicus. Gluteofemoral (GF) AT was collected 10–20 cm below the greater trochanter on the most lateral side of the upper thigh. Samples were cleaned at the bedside; a fraction of it was snap frozen in liquid nitrogen, a fraction of it was fixed overnight in Z-fix solution for future histology procedures and the rest was immediately used for stromal vascular fraction (SVF) isolation as described in [27]. Freshly isolated SVF cells were analyzed and sorted by a flow cytometric method as described below. The characteristics of the study group are presented in Table 1. One subject per group did not consent to the AT biopsies. FACS data were obtained for 10 control (BMI = 34.2 kg/m^−2^ ± 2.5 age = 33 years ± 6.2) and 4 PCOS (BMI = 34.7 kg/m^−2^ ± 3.1 age = 25 years ± 1.8) women. Four subjects per group were selected for RNA-seq analysis on their isolated ASCs. Blood samples were collected the day before the AT biopsies. Peripheral blood mononuclear cells (PBMCs) were isolated using Ficoll standard protocol (GE Healthcare, Chicago, IL, USA) and analyzed by flow cytometry as described below.

### 2.2. Body Composition and Biochemical Parameters

Body composition was measured by DXA (QDR 4500A, Hologic, Marlborough, MA, USA). Blood samples were processed and analyzed by routine clinical methods within 24 h of collection at the Adventhealth Laboratory at Orlando. The QUICKI index was calculated to evaluate insulin resistance [28].

### 2.3. Real-Time Quantitative RT-PCR

Total RNA from AT was isolated using an RNeasy Lipid Tissue Mini kit (Qiagen, Valencia, CA, USA). Primer-probe sets were pre-designed Single Tube Taqman^®^ gene expression assays. RT-qPCR reactions were performed using Taqman Fast Virus 1-step reaction mix Standard protocol (Life Technologies, Carlsbad, CA, USA). Data were normalized by using the internal control gene PPIA as previously described [29]. The 2^−∆∆CT^ method was used to analyze the relative changes in genes expression between groups and depots. The results are expressed in Arbitrary Units (AU).

### 2.4. Histology of Adipose Tissue Samples and Picrosirius Quantification

A fraction of AT biopsies was fixed and prepared for histologic analysis by using standard procedures. The adipocyte size (μm) was estimated by measuring the major diameter of 200 cells from digital microscopic images using NIS-Elements AR5.02 (Nikon, Minato, Japan) software. Picrosirius staining was quantified on one entire section for each sample using Halo software (Indicalabs, Albuquerque, NM, USA) and represented as the percentage of the total surface of the biopsy.

### 2.5. Culture of Human Adipose Tissue Explants

Adipokine secretions from paired ABD and GF AT from 18 subjects (13 control and 5 PCOS) were obtained. AT explants (100 mg) were incubated in duplicate in 1 mL of endothelial cell basal medium containing 1% bovine serum albumin, penicillin (100 U/mL), and streptomycin (100 mg/mL) under aseptic conditions. After 24 h incubation, supernatants were collected, centrifuged at 12,000× *g* for 10 min to remove cell debris, and stored at −80 °C until required. IL-6, adiponectin, IL-8, IL1-ra and MCP1 were measured by ELISA according to the manufacture protocol (Duoset ELISA kits, R&D systems, Minneapolis, MN, USA).

### 2.6. Flow Cytometry Analysis on Adipose Tissue and Blood Samples

ABD and GF freshly isolated SVF cells or PBMC fraction were incubated with fluorescent-conjugated antibodies or their respective controls for 30 minutes on ice in the dark. Then, cells were washed twice in cold PBS supplemented with 0.5% BSA and 0.2% EDTA. Data acquisition was performed using a BD FACS ARIA SORP cell sorter (FACSDiva software v5, BD Biosciences, San Jose, CA, USA) equipped with two lasers (blue 488 nm and red 640 nm). Compensation beads, isotype controls and fluorescent-conjugated antibodies were purchased from BD Biosciences (San Jose, CA, USA).

All antibodies were mouse monoclonal and specific against human cell surface markers. For SVF cells, fluorescent-conjugated antibodies were grouped in three analytical panels, which were carefully designed taking into account the cytometer configuration and the peculiarities of tissue cytometry. SVF-derived macrophages: anti-CD14 APC-C7 (clone MΦ P9, BD) and anti-CD206 APC (clone 19.2, BD); SVF-derived endothelial cells: anti-CD31 FITC (clone WM59, BD); SVF-derived preadipocytes: anti-CD34 PE-Cy7 (clone 581, BD) and anti-CD105 PerCP-Cy5.5 (clone 266, BD). For the blood samples, fluorescent-conjugated antibodies were added separately according to the following 2 panels: PBMC-derived monocytes: anti-CD14 APC-C7 (clone MΦ P9, BD); PBMC-derived endothelial progenitor cells: anti-CD34 PerCPCy5.5 (clone 8G12, BD) and anti-KDR PE (clone 89106, BD). Cell populations analyzed in this study were identified by the following patterns of surface markers. Preadipocytes: CD34+/CD105+/CD31dim; endothelial cells: CD34+/CD31+; macrophages: CD34-/CD14+/CD105+; monocytes: CD14+; EPCs: CD34+/KDR+. 

### 2.7. Isolation of Adipose-Derived Stem Cells (ASCs) and Culture Conditions

SFVs were isolated from fresh subcutaneous ABD and GF AT biopsies, plated, and grown as previously described [27] with the addition of hEGF and hFGF (Life Technologies, Carlsbad, CA, USA, 10 μg/mL and 4 μg/mL, respectively) during the expansion phase [30]. The ASC population was initially purified by depletion of the SVF from macrophages (CD14+/CD206+), endothelial cells (CD31+) and a subpopulation of preadipocytes (CD34+/CD105+) using fluorescence-activated cell sorting. At confluence, cells were harvested and frozen for future RNA-seq, DNA methylation analysis or in vitro differentiation as described in [27].

### 2.8. Lipolysis Assay

Glycerol accumulation in culture medium of fully differentiated ASCs (Day 11 of differentiation) was measured as an indicator of lipolysis. Basal lipolytic rates were defined as glycerol accumulation after 24 h and stimulated lipolysis was measured with isoproterenol (10^−6^ M) during 24 h. Glycerol concentration in culture media was measured with a standard enzymatic fluorometric assay (Sigma-Aldrich, St. Louis, MI, USA, cat#MAK117).

### 2.9. RNA-Seq

Total RNA was isolated using the RNeasy Mini Kit (Qiagen, Valencia, CA, USA) from ABD- and GF-ASCs of 4 control (BMI = 36.2 kg/m^−2^ ± 2.4 age = 28 years ± 4.7) and 4 PCOS (BMI = 35.9 kg/m^−2^ ± 3.2 age = 26 years ± 3.0) women. Libraires were prepared as described by Divoux, A. et al. [31] and sequenced on an Illumina HiSeq 2500 instrument. Raw data were aligned to the hg19 genome (GENCODE v19) using STAR v2.7.7a. SAMtools v1.3.1 was used for indexing and filtering the low-quality reads (MAPQ < 10). To quantify the transcriptome abundance and generate a raw read count table, Rsubread’s featureCounts v2.4.0 was used. Differentially expressed genes with a *p*-value < 0.05 were evaluated using by DESeq2 v1.30.1, an R package. Genes were filtered by expression. Counts per Million (CPM) were calculated and normalized with variance stabilizing transformation (vst). Heatmaps were generated by using ComplexHeatmap version 2.6.2, an R package. CPM values were transformed to z-score and the Ward.D2 method was used for row clustering.

### 2.10. DNA Methylation

Genome-wide methylation analysis was performed using the Illumina Infinium MethylationEPIC BeadChip platform (Illumina, San Diego, CA, USA), as described by Divoux, A. et al. [32]. Differentially methylated CpG sites between PCOS (*n* = 4, BMI = 35.9 kg/m^−2^ ± 3.2 age = 26 years ± 3.0) and control (*n* = 7, BMI = 34.7 kg/m^−2^ ± 2.5 age = 33 years ± 6.9) women were identified by ANOVA using FDR cutoff = 0.05 and estimated change in β-value cutoff ≥0.1 to define differentially methylated CpG sites. Analyses were performed independently in ABD- and GF-derived ASCs. CpG site annotation was based on MethylationEPIC_v-1-0_B4 beadchip from Illumina (species ‘Homo sapiens’, genome build hg19), using the UCSC database.

### 2.11. Gene Set Enrichment and Visualization

HypeR, an R package, was used for gene set enrichment and visualization [33]. The enrichment was calculated to the Hallmark gene set of the Molecular Signature Database (MSigDb). Significant pathways with *p*-value < 0.05 were selected.

## 3. Results

### 3.1. Body Composition and Hormone Levels

Clinical characteristics for PCOS and non-PCOS women who were matched for weight, BMI and WHR are shown in Table 1. All the women included in this study were apple shaped, meaning their WHR is greater than 0.8. Interestingly, although younger, PCOS women showed a significant lower level of total lean mass (Table 1). More detailed DXA results on body composition revealed no differences in arm fat, leg fat and lean masses between groups, whereas lower-body lean mass (gynoid) and the trunk lean mass (android) were lower in PCOS subjects compared to the controls (Figure S1). As to be expected, PCOS women exhibited higher testosterone levels (total, free and bioavailable; Table 1).

### 3.2. Gene Expression Profile of Abdominal and Gluteofemoral Adipose Tissue in PCOS and Non-PCOS Women

All women included in our study accumulated fat preferentially in the upper-body depots (see WHR in Table 1) and therefore have an “apple” body shape. Our original hypothesis posits that the lower-body AT depot in apple-shaped women is not able to appropriately accumulate lipids and our goal here was to extend this hypothesis to women with PCOS. Thus, we evaluated expression of genes related to lipid storage, inflammation, angiogenesis and extracellular matrix (ECM) in paired subcutaneous ABD and GF AT biopsies by RT-qPCR. Similar to what was observed in the control group with abdominal obesity, we identified a significant decrease in the GF in PCOS women compared to the ABD depot of genes related to lipid metabolism (*LPL*, *CD36*, *SNAIL* and *ADIPOQ*; Figure S2A), angiogenesis (*VEGFα*, *RSPO3*; Figure S2B) and perturbation of genes involved in ECM remodeling (*FN1*, *COL6A1* and *MMP3*; Figure S2C). Next, we estimated fibrosis accumulation in both AT depots by quantification of picrosirius staining in AT sections (Figure S2D). Interestingly, we found an increase in fibrosis in the GF relative to the ABD depot for both groups. Finally, we performed lipolysis assays following isoproterenol stimulation on in vitro differentiated ABD- and GF-ASCs and confirmed a decrease in lipolysis capacity in GF compared to ABD cells (Figure S2E), consistent with the literature [34]. Altogether, these results support our hypothesis that the GF AT depot of upper-body obese women is less prone to expand and accumulate lipids compared to their ABD AT. Importantly, these results are similar in control and PCOS groups.

**Figure 1:**
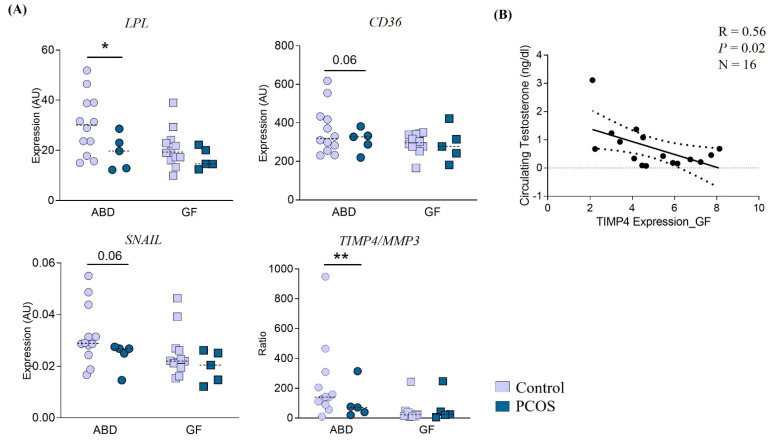
Effect of PCOS on abdominal (ABD) and gluteofemoral (GF) adipose tissue gene expression. (**A**) RT-qPCR analyses using primers against the genes listed were performed in ABD and GF adipose tissue in 12 control (light blue) and 5 PCOS (dark blue) women. The Mann–Whitney unpaired test was used. Dotted lines represent the median for each group. AU = Arbitrary Unit. * *p* ≤ 0.05 ** *p* ≤ 0.01. (**B**) Negative association between GF tissue expression of TIMP4 and circulating testosterone level.

We next hypothesized that the subcutaneous AT depots of PCOS women present more inflammation and fibrosis and less angiogenic capacity compared to the AT depots from non-PCOS apple-shaped women. We compared gene expression measured by RT-qPCR between the two groups of subjects, in each AT depot. Surprisingly, no differences were detected in the GF fat for the panel of candidate genes tested. The only significant differences in gene expression were observed in the ABD depot with a lower expression of *LPL*, *CD36* and *SNAIL* genes (involved in fatty acid metabolism) in PCOS (Figure 1A).

Finally, the ratio of expression *TIMP4/MMP3* was significantly lower in PCOS women compared to control (Figure 1A). TIMP4 is an activator of adipogenesis [35] and the ratio *TIMP4/MMP3* is thought to reflect a better adipogenesis capacity [36]. Because PCOS is associated with higher circulating testosterone (Table 1), which is thought to contribute to the disease phenotype, we evaluated the correlation between AT gene expression and circulating testosterone levels. This shows that *TIMP4* expression in GF depot negatively correlates with the level of testosterone in all subjects (Figure 1B).

### 3.3. Cellular Components of ABD and GF Adipose Tissue Depots in PCOS and Non-PCOS Women

**Figure 2:**
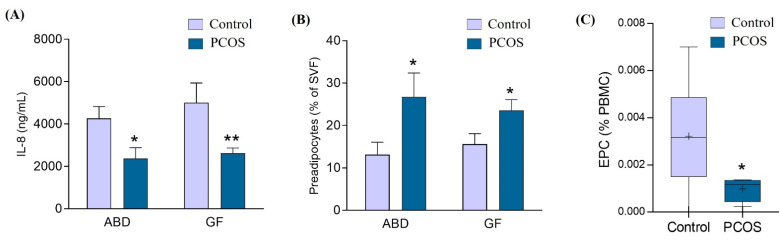
Effect of PCOS on abdominal (ABD) and gluteofemoral (GF) adipose tissue secretome and cellular content. (**A**) Conditioned medium was collected from 18 paired ABD and GF adipose tissue explants (13 control and 5 PCOS women) and the amount of IL-8 was measured with ELISA. The Mann–Whitney test was used. * *p* = 0.07 ABD control vs. ABD PCOS. ** *p* = 0.03 GF control vs. GF PCOS. (**B**) Freshly isolated stroma vascular fraction (SVF) cells from ABD (left) and GF (right) adipose tissues were analyzed by FACS. Preadipocytes (CD34+ CD105+ CD31dim) were quantified and reported as the percentage of total SVF cells. Data are from *n* = 10 control and 4 PCOS women. The Mann–Whitney test was used. * *p* ≤ 0.1. (**C**) Freshly isolated PBMCs from 11 control and 4 PCOS women were analyzed by FACS and the number of EPCs (CD34+ KDR+) was quantified. The Mann–Whitney test was used. * *p* ≤ 0.1.

To gain more insight into the potential roles for subcutaneous AT depots in the pathology of PCOS, we measured the secretion of inflammatory cytokines and chemokines (adiponectin, IL-6, IL-1ra, MCP-1 and IL-8) in ABD- and GF AT-derived explants. While there was lower IL-8 secretion by ABD and GF AT in PCOS compared to the control group, no other major differences were observed (Figure 2A). In addition to its role in inflammation, IL-8 is also an important regulator of angiogenesis in AT. This result suggests a default of vascularization in PCOS women. To further explore this hypothesis, we quantified by FACS the number of endothelial cells in the stroma vascular fraction (SVF) from ABD and GF AT biopsies of a subgroup of PCOS woman (*n* = 4) and control (*n* = 10). In addition to endothelial cells, SVF contains immune cells, preadipocytes and stem cells. Their number and/or phenotypes vary according to the degree of obesity and metabolic status [37]; however, to our knowledge, no study has reported their relative abundance in AT from PCOS women. Surprisingly, we did not observe changes in the number of endothelial cells in the subcutaneous fat depots between the two groups. The same result was found for the number of macrophages. However, we found an increase of 50% and 30% in preadipocytes from PCOS compared to the control group, in ABD and GF depots, respectively (Figure 2B), suggesting a role for adipose precursor cells in PCOS biology.

Finally, we measured the number of circulating endothelial cell progenitors (EPCs) which has been associated with the angiogenic status in a number diseases including T2D [38]. We first isolated the PBMCs from the blood of the participants and then determined the number of EPCs by FACS as CD34- and VEGFR2-positive cells. This revealed a decrease in EPCs in the blood of PCOS subjects (Figure 2C), which is consistent with an alteration of angiogenesis in PCOS women [39]. The lower secretion of IL-8 from both AT in PCOS may result in a more global defect of angiogenesis. Importantly, previous research showed an inversed correlation between EPC number and metabolic syndrome severity [40].

### 3.4. Comparison of Gene Expression Profiles in ASCs Isolated from ABD and GF Fat from Non-PCOS and PCOS Women Using RNA-Seq

**Figure 3:**
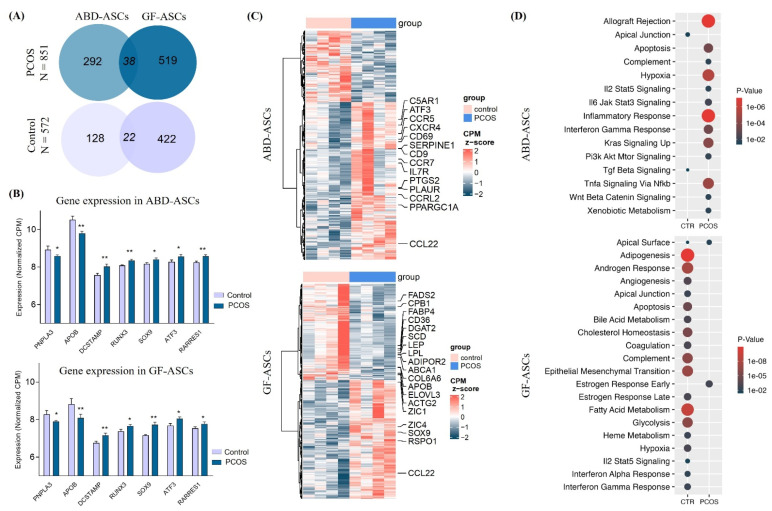
Effect of PCOS on global gene expression in abdominal (ABD)- and gluteofemoral (GF)-derived ASCs. Differentially expressed gene (DEG) analysis by RNA-seq between ASCs derived from control (*n* = 4) and PCOS (*n* = 4). (**A**) Genes were separated into up (dark blue) and down (light blue) regulation in PCOS vs. control samples. Venn diagrams show the overlap between DEGs found in ABD- and GF-derived ASCs for genes up (top) and down (bottom) regulated in PCOS women. (**B**) Representation of selected differentially expressed genes in control and PCOS women in ABD-ASCs (top) and in GF-ASCs (bottom). Average normalized CPM values are plotted for each group. * *p* < 0.05, ** *p* < 0.01. (**C**) Heatmaps show the DEGs in ABD-ASCs (left) and in GF-ASCs (right). Key genes are highlighted, and a comprehensive list is shown in Table S1. (**D**) Functional enrichment analysis performed by HypeR on the genes upregulated in PCOS vs. control in ABD-derived ASCs. Size represents the number of genes found in the respective pathway. Only pathways with *p* < 0.05 are represented.

**Figure 4:**
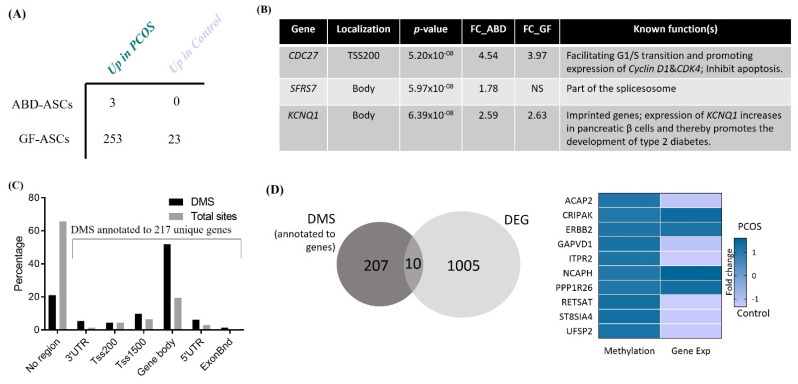
Effect of PCOS on global DNA methylation in abdominal (ABD)- and gluteofemoral (GF)-derived ASCs. DNA methylation was calculated as the average DNA methylation of each CpG sites on the Infinium Human Methylation 450 BeadChip in 7 control and 4 PCOS-derived ASCs. (**A**) Table shows the number of differential methylated sites (DMS) between PCOS and control ASCs, derived from ABD (top lane) and GF (bottom lane) fat depot. FDR < 0.05 and delta > 0.1. (**B**) List of the three genes associated with hypermethylated sites in PCOS women found in ABD-derived ASCs. NS: not significant. (**C**) Distribution of significant methylated sites (grey) compared with all analyzed sites (black) in relation to nearest gene region. TSS (Transcriptional Start Site), proximal promotor defined as 200 or 1500 bp upstream of the transcription site. DMS: Differentially Methylated Site. (**D**) Venn diagrams show the overlap between differentially expressed genes (DEGs) and differentially methylated sites annotated to the nearest gene (DMS). Heatmap represents the fold change of methylation (left) and expression (right) between PCOS and control GF-derived ASCs for the 10 overlapping genes.

Adipose progenitor cells play a central role in depot-specific AT expansion through proliferation and differentiation capacity [41,42]. A failure of proper differentiation could lead to IR reflected by the presence of large adipocytes filled with excess lipid and enhanced inflammatory markers [43]. To dissect the molecular mechanisms and the affected gene pathways accompanying the increase in preadipocyte infiltration observed in both subcutaneous fat tissue in PCOS, we performed RNA-seq analysis in ASCs isolated from ABD and GF AT biopsies of four PCOS and four control group women, matching for BMI and age (see Methods). We identified 851 genes upregulated in PCOS compared to the control group, 292 and 519 genes being found only in ABD- and GF-derived ASCs, respectively (Figure 3A). We observed 572 genes downregulated in PCOS sample—128 and 422 genes in ABD- and GF-derived ASCs, respectively. Sixty-two differentially expressed genes (DEGs) between PCOS and the control groups were found simultaneously in cells derived from both depots—38 genes upregulated, 22 genes downregulated in PCOS and 2 genes showing opposite regulation in ABD and GF cells (Figure 3A, intersection of the Venn diagrams, full list in Table S1). Importantly, *PNPLA3* (coding for Adiponutrin) and *APOB*, both associated with lipid metabolism, were downregulated in PCOS-derived cells from both AT (Figure 3B). Genes involved in osteoblast differentiation (*DCSTAMP* and *RUNX3*), early stem cell lineage (*SOX9*) and genes that could inhibit adipogenesis (*ATF3* [44] and *RARRES1* [45]) were upregulated in PCOS cells isolated from both AT (Figure 4B). Collectively, these results indicate that the ASCs derived from the control women may be more committed to adipocyte lineage than the precursor cells derived from the PCOS subjects.

Figure 3C depicts the heatmap of the DEGs between PCOS and control in ABD-derived ASCs (top) and GF-derived ASCs (bottom); for the full list of DEGs, see Table S1. Genes involved in inflammation (*CCL22*, *C5AR1*, *CCR5*, *CXCR4*, *CCR7*, *IL7R*, *PTGS2*, *PLAUR*, *CCRL2*, *SERPINE1* and *CD69*) are specifically upregulated in PCOS ABD-ASCs compared to the control stem cells. Importantly, *CD9*, recently identified as a marker of profibrotic and poorly adipogenic cells in human visceral AT [46], was also upregulated in PCOS ABD-ASCs. In contrast, genes involved in lipid storage and lipid droplet formation are downregulated in PCOS vs. control cells derived from GF AT (*FADS2*, *CPB1*, *FABP4*, *CD36*, *DGAT2*, *SCD*, *LEP*, *LPL*, *ADIPOR2*, *ABCA1*, *ELOVL3*, and *ACTG2*).

To further gain insight into gene function, we performed a gene-enrichment analysis with the DEGs in cells derived from each fat depot. Interestingly, the most enriched pathways in ABD PCOS cells were allograft rejection (regrouping immune system genes), inflammatory response, TNFα signaling via NFκB, complement, and hypoxia (Figure 3D, top); all of which are likely to be associated with impaired adipogenic capacity [47,48]. In contrast, the differentially enriched pathways in GF are mainly observed in control cells (Figure 3D, bottom), the most significant pathways being involved in adipocyte development (adipogenesis, cholesterol homeostasis, fatty acid metabolism and glycolysis, for example). Altogether, these data suggest that ASCs derived from PCOS are more stem like and express fewer genes involved in lipid storage, notably in GF AT. Furthermore, ABD-ASCs isolated from PCOS are more pro-inflammatory compared to the control ABD precursor cells consistent with an increased potential for metabolic disease progression.

### 3.5. Hypermethylation in GF-ASCs Derived from PCOS Women

Emerging evidence from human studies and mouse models suggests that maternal androgen excess before and shortly after birth predisposes the next generation to PCOS [49]. Importantly, a recent study reported that differentially methylated signatures in the ovaries of PCOS-like mice were transmitted to their third-generation offspring, suggesting a stable pattern of epigenetic transmittance. Interestingly a similar signature was also found in blood cells from daughters born to women with PCOS [50], suggesting that epigenetic programming relevant to PCOS occurs during development. Only a few studies have investigated epigenetic modifications in PCOS humans—most focus on DNA methylation in ovarian tissue [51,52], granulosa cells [53,54] and in blood samples [55].

To investigate the effect of PCOS on ABD- and GF-derived ASC DNA methylation profiles, we used Illumina Infinium HumanMethylation450k array BeadChips. Importantly, PCOS and control groups were matched for BMI and age (see Section 2.10. Methylation status was obtained for 483,317 CpG sites. We identified only three differentially methylated sites (DMS) between PCOS and control ASCs derived from the ABD fat depot, all of them being hypermethylated in PCOS (FDR < 0.05—Figure 4A). Figure 4B shows the nearest gene found next to those DMS. Interestingly, *KCNQ1* is a susceptible gene for T2D [56]. Moreover, *KCNQ1* hypermethylation in pancreatic beta cells was shown to associate with higher gene expression [57] and therefore decreased insulin secretion. The role of KCNQ1 in the context of PCOS and potential implication in higher risk to develop T2D merits further investigation.

In contrast to the relatively low level of DMS in ABD-derived ASCs, 276 DMS were found in GF cells. A total of 253 were hypermethylated in PCOS and 23 were hypermethylated in control (FDR < 0.05—Figure 4A—full list in Table S2). The average levels of DNA methylation for these sites were grouped based on their location in relation to the nearest gene (Figure 4C) or in relation to CpG islands (Figure S3) and compared to the total number of sites on the array. Interestingly, we found an enrichment of the DMS in gene body regions (more than 50% of the DMS), whereas the majority of the total methylation sites are localized in non-gene-associated regions (Figure 4C). Those sites were annotated to 217 unique genes. The most significant CpG sites were cg1582158 and cg0187731, annotated to *FUBP1* and *CDC27*, respectively (Fold change 12 and 4 PCOS vs. Control). FUBP1 promotes cell proliferation and enhances cell migration [58]. CDC27 facilitates G1/S transition during cell cycle and promotes expression of *Cyclin D1* and *CDK4* genes. CDK4 promotes adipogenesis through PPARγ activation and is an essential insulin effector in adipocytes [59,60]. Both proteins inhibit apoptosis. Thus, these genes are known to affect key pathways relevant to AT function; however, their specific role in AT biology or adipogenesis has not yet been explored.

To assess a broader biological relevance for the differently methylated genes in GF-ASCs from women with PCOS and controls, we used pathway analysis (see Section 2.11) to analyze the 217 genes annotated to the DMS. The two significantly upregulated canonical pathways were mitotic spindle (*ARAP3*, *CDC27*, *FGD6*, *PCM1*, *PLEKHG2*, and *TSC1*), responsible for accurate chromosomes segregation during mitotic division, and allograft rejection (*BCAT1*, *EGFR*, *JAK2*, *LTB*, and *ST8SIA4*), *p*-value 0.0054 and 0.023, respectively. Importantly, BCAT1, EGFR and JAK2 are required for cell proliferation signaling in cancer cells [61,62] or preadipocytes [63]. These results indicate a dysregulation of GF-ASC proliferation rate in PCOS women and are consistent with an alteration of mesenchymal stem cell proliferation recently observed in a rat model of PCOS [64]. During early adipogenesis, cells undergo a strong downregulation of translational activity with a decrease in cell size, proliferation and migration. Collectively, our data revealed hypermethylation near genes involved in cell cycle and cell proliferation in PCOS GF-ASCs, suggesting a modification of adipogenesis capacity in PCOS women.

Finally, to explore the influence of higher circulating testosterone in PCOS women, on the level of DNA methylation in AT, we performed a correlation analysis between DNA methylation level in GF-ASCs and circulating testosterone level in our complete set of control and PCOS women. We identified 146 genes with a significant correlation between the 2 factors (Spearman’s correlation, *p* < 0.05; Table S3). Importantly, the level of methylation for 135 genes was positively associated with testosterone concentration, including FUBP1 and genes involved in cell cycle processes (*CDC27*, *CHMP6*, *HERC2*, *JAK2*, *NCAPG2*, *NUP54*, *PCM1*, *PTTG1*, *RAD17*, and *TNPO1*). One of the strongest correlations was found for *NLRP1* (R = 0.83—Table S3), which is a key mediator of the inflammasome and is associated with obesity and metabolic syndrome [65].

### 3.6. Association between Differentially Expressed and Methylated Genes in GF-ASCs

We next investigated the correlation between the DEGs and DMS between PCOS and control groups. We overlapped the list of DEGs in GF-ASCs and the list of genes annotated to DMS in GF-ASCs. We found 10 genes with both significant differences in expression and DNA methylation (Figure 4D). Six were downregulated when the respective CpG site is hypermethylated, suggesting a classic mechanism of transcriptional repression by DNA methylation. Importantly, the *ERBB2* (HER2) gene, a key factor in cell proliferation in cancer but also involved in adipocyte differentiation [66], is simultaneously hypermethylated at its 5′ UTR and overexpressed in PCOS cells compared to control (Figure 4D). This is interesting because DNA methylation regulates gene expression and has historically been linked to gene repression. However, recently, its specific role in gene activation has been identified and new data have revealed a broader role for DNA methylation in gene expression, both positively and negatively, through sites included in both promoter and non-promoter regions [67]. Our results are consistent with this broader role for DNA methylation in the regulation of gene expression.

## 4. Discussion

Data from earlier studies, including work from our lab [25,32,68], revealed distinct transcriptional and methylome signatures between ABD and GF ATs and between apple- and pear-shaped women. Here, to our knowledge, we report the first comparison of upper and lower-body adipose biopsies between obese PCOS and BMI/WHR-matched control women. Consistent with the findings in apple- vs. pear-shaped non-PCOS women, we found that ABD AT of PCOS and control apple-shaped women appeared prone to better expansion compared to their GF AT. More specifically, we observed an increase in expression of genes involved in lipid metabolism, angiogenesis and ECM remodeling in ABD AT, all these processes being interconnected during AT growth. In addition to the depot-specific differences, we also identified key traits related to the PCOS phenotype. Surprisingly, these differences were observed mainly in ABD AT, where a defect in lipid accumulation in PCOS is suggested by decreased *LPL*, *CD36*, *SNAIL* and the ratio of *TIMP4/MMP3* gene expression. A recent study in mice showed that under a high-fat diet, the absence of Timp4 leads to reduced AT hypertrophy and fibrosis compared to the wild-type counterparts [69]. This is consistent with a reduced capacity for lipid deposition in the ABD AT of PCOS, leading to more accumulation of lipids in non-adipose peripheral tissues such as liver, VAT and pancreas and overall leading to metabolic syndrome.

Interestingly, we observed a decrease in IL-8 secretion in PCOS AT explants. IL-8 is a major angiogenic factor secreted by human AT, suggesting a defect of vascularization in PCOS fat depot. Our data show that hypoxia is one of the main pathways upregulated in PCOS ABD-derived ASCs, which could be the direct consequence of lack of oxygenation in PCOS tissue. This is consistent with earlier work from our lab using direct measurement of AT partial pressure of oxygen [70]. Interestingly, we also observed a decrease in endothelial precursor cells (EPCs) in the blood of PCOS women. A decrease in these progenitors could result in an impaired epithelium damage repair, which could contribute to the increased risk of cardiovascular disease in PCOS subjects [71].

Resident preadipocyte proliferation and differentiation are key to AT expansion. A reduction in adipogenesis capacity or an increase in differentiation inhibitory signals to preadipocytes could result in the failure of proper AT growth coupled with the appearance of metabolic complications associated with obesity [19,20,72]. We observed an increase in the number of preadipocytes in both ABD and GF subcutaneous AT depots of PCOS subjects. This is a novel observation suggesting active remodeling of AT in PCOS. We then isolated the precursor cells in each subcutaneous AT and characterized their unique transcriptional and methylome signatures. We identified two very distinct transcriptional profiles according to the origin of the precursor cells. ABD-derived ASCs showed an enrichment for inflammatory markers in PCOS women compared to the control non-PCOS apple-shaped group. Several studies have shown that the pro-inflammatory environment inhibits preadipocyte differentiation and leads to IR [73,74]. Interestingly, the Pi3k/Akt/Mtor pathway, known to be stimulated by insulin, and the Wntβ pathway, an inhibitor of adipogenesis [75], are also upregulated in ABD-ASCs derived from PCOS women. The Pi3k/Akt/Mtor pathway influences stem cell proliferation and may inhibit adipogenesis when activated [76]. In addition, this pathway is regulated by the androgen receptor in prostate cancer cells [77], suggesting that the hyperandrogenism characteristic of PCOS could modulate the gene expression and phenotype of ABD-derived ASCs.

In the lower-body fat depot, we observed a defect in lipid storage capacity in the PCOS vs. the control women. This is similar to the reduced lipid storage gene expression we see in apple- vs. pear-shaped women-derived adipocytes [25]. The GF-ASCs derived from the apple control group were enriched for genes involved in adipogenesis, fatty acid metabolism and glycolysis compared to the GF-ASCs derived from PCOS. These observations are surprising knowing that control and PCOS women are apple shaped and have a limited amount of lower-body fat. The GF adipose stem cells of PCOS appear even further stunted in their ability to differentiate into mature adipocytes compared to the apple control group, who already have a limited capacity for AT expansion in comparison to pear-shaped women. Understanding the origin of such a difference could help us understand why a large majority of obese PCOS women are apple shaped and have higher risks of developing metabolic syndrome.

We further studied the methylome of ASCs derived from ABD and GF and identified hypermethylated CpG sites almost exclusively in GF-ASCs of PCOS women. These epigenetic marks are associated with a fraction of the differential genes observed in the GF precursor cells between PCOS and control. Notably, we identified DMS at proximity of genes related to cell cycle, cellular proliferation and transcription factors (Table S2). The level of methylation of genes related to cell cycle were also correlated with the circulating level of testosterone, hyperandrogenemia being observed in most PCOS women. The transition between cell proliferation and cell differentiation taking place during adipocyte differentiation is a tightly regulated process, where both cell cycle regulators and differentiating factors interact, creating a cascade of events leading to the commitment of the cells into the adipocyte phenotype [78]. More specifically, adipose precursor cells need to go through clonal expansion, where the mitotic spindle is required, before they start differentiation [79]. Our data suggest that key mitotic differential methylation status between PCOS and control could influence the expression of cell cycle genes and transcription factors involved in stem cell commitment to the adipogenic lineage.

Altogether, our data suggest a defect of adipogenesis in precursor cells isolated from PCOS women. That could result in lower capacity of subcutaneous AT expansion and explain the elevated metabolic disease risk associated with PCOS. The increase in preadipocytes observed in PCOS AT could be a consequence of the reduction in their capacity to differentiate into adipocytes. Additional in vivo experiments using, for example, deuterium-labeled water followed by AT biopsy to measure the incorporation of ^2^H into the DNA of newly formed adipocytes [80] could help to determine the conversion of preadipocytes into adipocytes within ABD and GF AT of PCOS women and compare with control women.

## 5. Conclusions

In summary, our exploratory analysis links for the first time PCOS with the AT expansion theory. This model stipulates that once the limit of growth of the subcutaneous AT is reached, lipids accumulate in other tissues, resulting in metabolic disease. Here, we extend this theory to the lower-body AT in PCOS women. Our results suggest a defect of adipogenesis of the GF-ASCs in PCOS women, compared to similarly apple-shaped non-PCOS women, in parallel with a more pronounced pro-inflammatory state of their ABD-ASCs. We observed an enrichment of hypermethylation sites in the DNA of PCOS women, only in GF-derived ASCs, at key genes involved in ASC function (i.e., proliferation) which could potentially contribute to the failure in lipid storage capacity in their defective GF depot. Collectively, our data suggest that the GF AT in PCOS women is programmed to lower expansion compared to the GF AT of apple control women. Importantly, the components of this program were correlated with testosterone. The influence of hyperandrogenism on AT transcriptional and epigenetic profile merits further investigation. In addition, the limited number of subjects used in this first exploratory study would need to be expanded to confirm the role of ASCs in PCOS etiology.

## Figures and Tables

**Table 1:** Clinical parameters of control and PCOS subjects (mean ± standard deviation).

Clinical Parameters	Control Subjects (*n* = 13)	PCOS Subjects (*n* = 6)	*p-*Value
Age (years)	34 ± 5.6	26 ± 2.7	0.008
Race (C/H/AA/other)	8/2/2/1	4/2/0/0	
Adiposity markers			
BMI (kg/m²)	34.5 ± 2.7	34.8 ± 3.1	0.95
Weight (kg)	96 ± 9.3	92 ± 6.1	0.41
Hip circumference	114.9 ± 7.19	116.9 ± 6.40	0.76
Waist circumference	107.1 ± 5.56	104.3 ± 2.92	0.25
Waist to hip ratio	0.93 ± 0.04	0.89 ± 0.04	0.15
Total fat mass (kg)	42.2 ± 6.91	44.5 ± 6.60	0.70
Total lean mass (kg)	52.1 ± 4.20	47.5 ± 3.94	0.05
Fat mass (%)	44 ± 3.7	48 ± 4.4	0.12
Lean mass (%)	54 ± 3.5	51 ± 3.8	0.13
Visceral fat mass (kg)	12.3 ± 4.33	10.4 ± 3.08	0.32
Energy expenditure (kcal/day)	2559 ± 300	2469 ± 304	NS
Metabolic markers			
Fasting glucose (mg/dL)	93.2 ± 11.6	85.8 ± 5.92	NS
Fasting insulin (mU/L)	5.49 ± 3.09	4.14 ± 1.74	NS
QUICKI	0.38 ± 0.05	0.39 ± 0.04	NS
FFA (mmol/L)	0.41 ± 0.09	0.51 ± 0.12	NS
Circulating Hormones			
TSH (mU/L)	1.78 ± 0.65	1.33 ± 0.81	NS
Total testosterone (ng/dL)	25.6 ± 17.6	63.2 ± 25.1	0.007
SHBG (nmol/L)	35.4 ± 21.1	51.8 ± 49.2	NS
Free testosterone (ng/dL)	0.46 ± 0.40	1.24 ± 0.91	0.05
Bioavailable testosterone (ng/dL)	11.5 ± 10.1	30.0 ± 22.4	0.05

NS = not significant.

## Data Availability

All sequencing data have been deposited to the NCBI GEO database (http://www.ncbi.nlm.nih.gov/geo/, accessed on 17 January 2022) under accession number GSE#193812.

## Supplementary Materials

The following supporting information can be downloaded at: https://www.mdpi.com/article/10.3390/cells11050848/s1, Table S1: List of differentially expressed genes between four control and four PCOS women in ABD-derived ASCs and in GF-derived ASCs. DESeq2, *p*-value < 0.05. The third list is the intersection of the first two lists and shows the common DEGs in ABD- and GF-derived ASCs. Table S2: List of differentially methylated sites between seven control and four PCOS women in ABD-derived ASCs and in GF-derived ASCs. ANOVA, FDR < 0.05 (ABD βvalue) GF βvalue) > 0.1. Table S3: List of genes annotated to CpG sites with level of methylation correlated to the level of circulating testosterone. Spearman’s correlation, *p*-value < 0.05, *n* = 10. Figure S1: Histograms show fat (**A**) and lean (**B**) masses measured by DXA in control (*n* = 13, light blue) and PCOS (*n* = 6, dark blue) women according to body regionality. Data are presented as the percentage of fat mass on total mass. The Mann–Whitney unpaired test was used. Figure S2: Gene expression profile of abdominal (ABD) and gluteofemoral (GF) adipose tissue in control and PCOS women. (**A**-**C**) RT-qPCR analyses using primers against the genes listed were performed in ABD and GF adipose tissue in 12 control (light blue) and 5 PCOS (dark blue) women. The Wilcoxon paired test was used. AU = Arbitrary Unit. (**D**) Levels of fibrosis were quantified in picrosirius red-stained slides and presented as percent of stained to total area (% total surface). *n* = 12 control and 5 PCOS women. The Wilcoxon paired test was used. (**E**) Paired ABD- and GF-ASCs were differentiated into adipocytes and glycerol release was measured on day 11, with or without isoproterenol stimulation. Data are presented as the fold change between glycerol release after stimulation compared to basal release. Each symbol represents ASCs derived from different donors (control in light blue and PCOS in dark blue). The Wilcoxon paired test was used. ** *p* < 0.01. Figure S3: Distribution of significant methylated sites compared with all analyzed sites in relation to nearest CpG island region. Shore, flanking region of CpG island (0–2000 bp). Shelf, regions flanking island shores (2000–4000 bp from the CpG island).
