# Supplementary material for: Transcriptional and DNA Methylation Signatures of Subcutaneous Adipose Tissue and Adipose-Derived Stem Cells in PCOS Women
Source: Cells. 2022 Mar 1;11(5):848. doi: 10.3390/cells11050848 (PMC8909136; doi:10.3390/cells11050848)
Supplement: Supplementary file 1 [file cells-11-00848-s001.zip › cells-1580887 supplementary proof back/cells-1580887 supplementary conversion.pdf]

Supplementary Materials:

Table S1: List of differentially expressed genes between four control and four PCOS women in ABD-derived ASCs and in GF-derived ASCs. DESeq2, *p*-value < 0.05. The third list is the intersection of the first two lists and shows the common DEGs in ABD- and GF-derived ASCs.

Table S2: List of differentially methylated sites between seven control and four PCOS women in ABD-derived ASCs and in GF-derived ASCs. ANOVA, FDR < 0.05 (ABD βvalue)-(GF βvalue) > 0.1.

Table S3: List of genes annotated to CpG sites with level of methylation correlated to the level of circulating testosterone. Spearman's correlation, *p*-value < 0.05, n = 10.

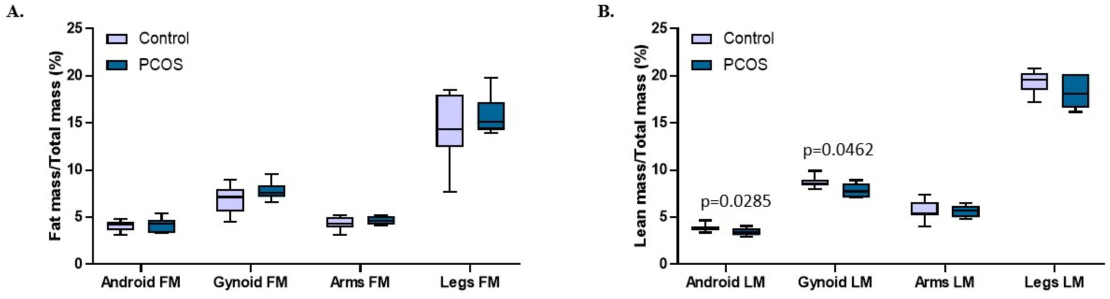

Figure S1. Histograms show fat (**A**) and lean (**B**) masses measured by DXA in control (n = 13, light blue) and PCOS (n = 6, dark blue) women according to body regionality. Data are presented as the percentage of fat mass on total mass. The Mann–Whitney unpaired test was used.

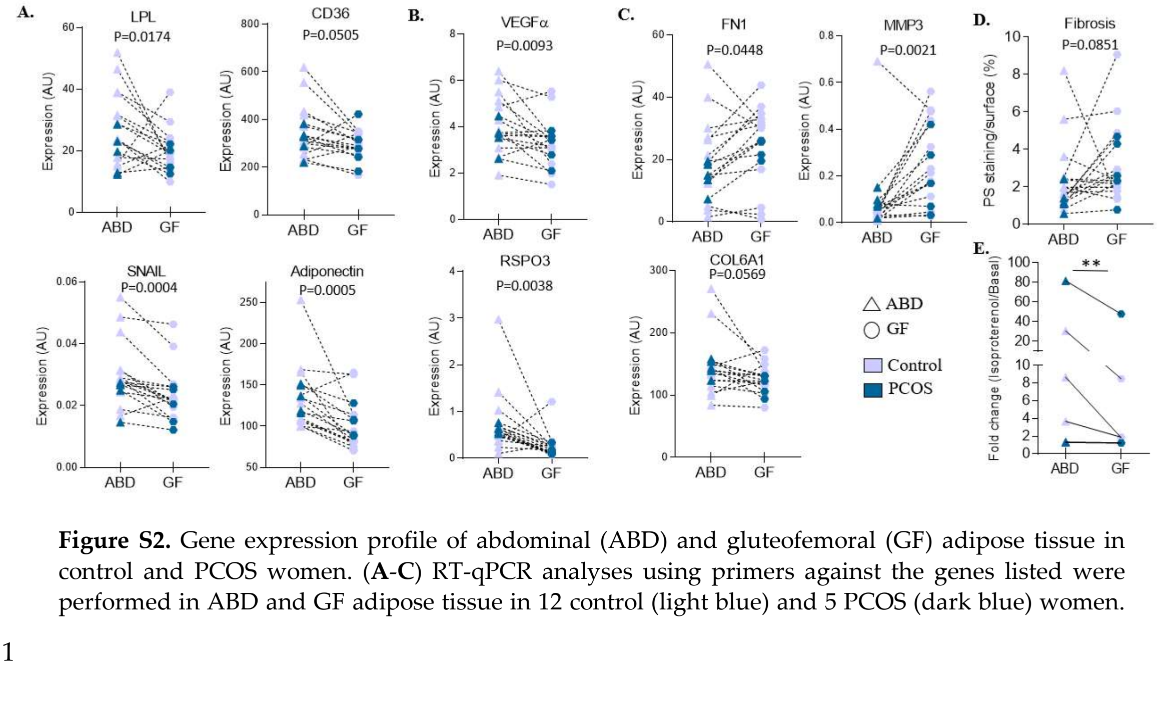

Figure S2. Gene expression profile of abdominal (ABD) and gluteofemoral (GF) adipose tissue in control and PCOS women. (**A**-**C**) RT-qPCR analyses using primers against the genes listed were performed in ABD and GF adipose tissue in 12 control (light blue) and 5 PCOS (dark blue) women.

The Wilcoxon paired test was used. AU = Arbitrary Unit. (**D**) Levels of fibrosis were quantified in picrosirius red-stained slides and presented as percent of stained to total area (% total surface). n = 12 control and 5 PCOS women. The Wilcoxon paired test was used. (**E**) Paired ABD- and GF-ASCs were differentiated into adipocytes and glycerol release was measured on day 11, with or without isoproterenol stimulation. Data are presented as the fold change between glycerol release after stimulation compared to basal release. Each symbol represents ASCs derived from different donors (control in light blue and PCOS in dark blue). The Wilcoxon paired test was used. ** $p < 0.01$.

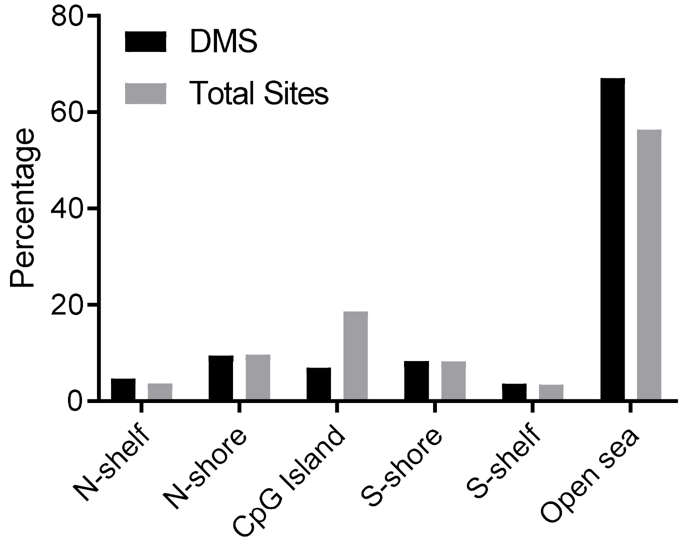

Figure S3. Distribution of significant methylated sites compared with all analyzed sites in relation to nearest CpG island region. Shore, flanking region of CpG island (0–2000 bp). Shelf, regions flanking island shores (2000–4000 bp from the CpG island).
